# High-flow nasal oxygen cannula vs. noninvasive mechanical ventilation to prevent reintubation in sepsis: a randomized controlled trial

**DOI:** 10.1186/s13613-021-00922-5

**Published:** 2021-09-14

**Authors:** Surat Tongyoo, Porntipa Tantibundit, Kiattichai Daorattanachai, Tanuwong Viarasilpa, Chairat Permpikul, Suthipol Udompanturak

**Affiliations:** 1 Division of Critical Care Medicine, Department of Medicine, Faculty of Medicine, Siriraj Hospital, Mahidol University, No. 2, Prannok Road, Bangkoknoi, Bangkok, 10700 Thailand; 2 Department of Emergency Medicine, Khon Kaen Hospital, Khon Kaen, Thailand; 3 Department of Emergency Medicine, Faculty of Medicine, Thammasat University, Pathum Thani, Thailand; 4 Office of Research and Development, Faculty of Medicine, Siriraj Hospital, Mahidol University, Bangkok, Thailand

**Keywords:** High flow nasal cannula, Non-invasive mechanical ventilation, Sepsis, Extubation, Extubation failure, Reintubation

## Abstract

**Background:**

High-flow nasal oxygen cannula (HFNC) and noninvasive mechanical ventilation (NIV) can prevent reintubation in critically ill patients. However, their efficacy in post-extubated sepsis patients remains unclear. The objective of this study was to compare the efficacy of HFNC vs. NIV to prevent reintubation in post-extubated sepsis patients.

**Methods:**

We conducted a single-centre, prospective, open-labelled, randomised controlled trial at the medical intensive care unit of Siriraj Hospital, Mahidol University, Bangkok, Thailand. Sepsis patients who had been intubated, recovered, and passed the spontaneous breathing trial were enrolled and randomly assigned in a 1:1 ratio to receive either HFNC or NIV support immediately after extubation. The primary outcome was rate of reintubation at 72 h after extubation.

**Results:**

Between 1st October 2017 and 31st October 2019, 222 patients were enrolled and 112 were assigned to the HFNC group and 110 to the NIV group. Both groups were well matched in baseline characteristics. The median [IQR] age of the HFNC group was 66 [50–77] vs. 65.5 [54–77] years in the NIV group. The most common causes of intubation at admission were shock-related respiratory failure (57.1% vs. 55.5%) and acute hypoxic respiratory failure (34.8% vs. 40.9%) in the HFNC and NIV groups, respectively. The duration of mechanical ventilation before extubation was 5 [3–8] days in the HFNC group vs. 5 [3–9] days in the NIV group. There was no statistically significant difference in the primary outcome: 20/112 (17.9%) in the HFNC group required reintubation at 72 h compared to 20/110 (18.2%) in the NIV group [relative risk (RR) 0.99: 95% confidence interval (CI) (0.70–1.39); *P* = 0.95]. The 28-day mortality was not different: 8/112 (7.1%) with HFNC vs. 10/110 (9.1%) with NIV (RR 0.88: 95% CI (0.57–1.37); *P* = 0.59).

**Conclusions:**

Among sepsis patients, there was no difference between HFNC and NIV in the prevention of reintubation at 72 h after extubation.

*Clinical Trial Registration* ClinicalTrials.gov Identifier: NCT03246893; Registered 11 August 2017; https://clinicaltrials.gov/ct2/show/NCT03246893?term=surat+tongyoo&draw=2&rank=3

**Supplementary Information:**

The online version contains supplementary material available at 10.1186/s13613-021-00922-5.

## Background

Extubation failure is a serious clinical event associated with a poor outcome in patients that have recovered from respiratory failure [1, 2]. The reported incidence range of reintubation after planned extubation is between 10 and 20%, depending on patient characteristics, weaning modality, and the follow-up period [3–5]. The risk of death is up to fivefold higher than in patients who do not experience extubation failure [3–7]. In addition, reintubated patients experience longer stays in the intensive care unit (ICU) and the hospital [8].

Previous studies have assessed measures that could prevent reintubation. Noninvasive mechanical ventilation (NIV) provides intermittent positive pressure with high and adjustable oxygen concentrate airflow and has been proven to decrease the risk of reintubation, especially in hypercapnic patients [9–12]. Guidelines from the European Respiratory Society and the American Thoracic Society recommend that NIV should be used to prevent reintubation in at-risk patients including patients age > 65 years and those with underlying cardiac or chronic respiratory disease [13]. High-flow nasal oxygen cannula (HFNC) is a more recent modality that provides heated, humidified air with an adjustable oxygen concentration via a wide-bore nasal cannula. A large-scale randomised controlled trial (RCT) comparing HFNC with conventional oxygen therapy after extubation in low-risk patients reported that HFNC was associated with a lower rate of reintubation at 72 h [14]. A recent RCT in critically ill and post-cardiothoracic surgery patients at high risk of extubation failure, reported that HFNC was not inferior to NIV [15, 16]. Moreover, an observational study showed that HFNC was as effective as NIV in preventing reintubation with a lower rate of device intolerance [17].

Despite these encouraging studies, there are no studies that conclude that either HFNC or NIV is superior to prevent reintubation, particularly in recovered sepsis patients. Approximately 80% of sepsis/septic shock patients experience respiratory failure and require mechanical ventilation [18, 19]. The in-hospital mortality of these patients ranges from 28% to 51.2%, which is higher than that in patients without respiratory failure [20–22]. Moreover, when patients improve and are extubated, approximately 19% experience extubation failure [23]. The high proportion of extubation failure among sepsis patients may be explained by fluid overload following septic shock resuscitation, sepsis related cardiomyopathy, and deterioration of kidney function [24, 25]. These may result in extubation failure due to weaning-induced pulmonary edema [26]. Therefore, we compared HFNC vs. NIV to prevent extubation failure among sepsis patients.

## Methods

### Study design

**Fig. 1:**
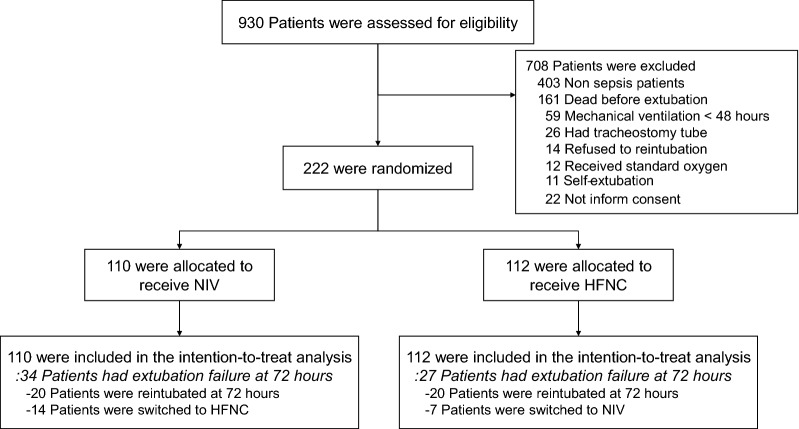
Flow diagram illustrating the screening, enrolment, and randomisation of patients HFNC: High-flow nasal oxygen cannula; NIV: noninvasive mechanical ventilation

This study was a prospective, randomised, unblinded clinical trial at two medical ICU in Siriraj Hospital, Mahidol University, Bangkok, Thailand between October 1, 2017 and October 31, 2019. The protocol was approved by the Siriraj Institutional Review Board (certificate of approval no. Si212/2017) and was conducted under the ethical principles of the Declaration of Helsinki. Prior informed consent for participation was obtained from patients or their legal guardians if the patient was unable to provide consent. All participant screening and enrolment procedures were performed by the coinvestigators (Fig. 1). The outcome evaluation data analysis was conducted by the principal investigator and a statistician, both of whom were blinded to the patient’s treatment group. The trial was funded by the Siriraj-Critical-Care-Research-Funding. The funder had no role in the study design, data analysis, or outcome assessment.

### Participants

All patients aged 18 years or older who were admitted to the medical ICU, intubated, and ventilated for at least 48 h were assessed for eligibility. Those who met the diagnostic criteria of sepsis or septic shock according to the Surviving Sepsis Campaign: International Guidelines for Management of Severe Sepsis and Septic Shock 2016(SEPSIS-3) [1] and then recovered from their critical condition were eligible for enrolment. Patients who underwent tracheostomy or unplanned extubation, and those who signed a “do not resuscitate” order were excluded. We classified acute respiratory failure requiring intubation into four categories based on the underlying pathophysiology. The categories were type 1) acute hypoxic respiratory failure, type 2) acute hypercapnic respiratory failure, type 3) acute respiratory failure caused by perioperative atelectasis, and type 4) acute respiratory failure during shock or hypoperfusion [27].

After obtaining informed consent, we performed daily assessments of the patients’ weaning readiness in accordance with a standard weaning protocol. Weaning readiness was defined according to the following criteria: (i) recovery from the current critical illness (temperature < 38 °C, mean arterial blood pressure ≥ 65 mmHg without vasoactive drugs or only low doses of dopamine [< 5 μg/kg/min] or norepinephrine [< 0.05 μg/kg/min], and heart rate < 120 beats/minute); (ii) rapid shallow breathing index < 105 breaths/min/L; (iii) partial pressure of oxygen in arterial blood to fraction of inspired oxygen (PaO_2_:FiO_2_ ratio) > 150 with FiO_2_ ≤ 0.4, positive end expiratory pressure (PEEP) < 8 cmH_2_O, and arterial pH > 7.35; (iv) no symptoms and electrocardiographic signs of active myocardial ischaemia; (v) haemoglobin level > 7 g/dL; and (vi) Richmond Agitation-Sedation Scale (RASS) score ≥ − 1 without neuromuscular blocking agent use in the last 12 h. Patients who fulfilled these criteria for weaning readiness underwent a spontaneous breathing trial with either a T-piece or pressure support of 8 cmH_2_O in accordance with their attending physicians’ judgement, for at least 30 min. Patients were considered to have failed the spontaneous breathing trial if they developed agitation, altered mental status, cyanosis, respiratory rate > 35 breaths/minutes, oxygen saturation < 90%, PaCO_2_ > 50 mmHg or a more than 8 mmHg increase from baseline value, heart rate > 140 beats/minute, systolic arterial pressure > 180 mmHg or an increase of more than 20% from baseline value, systolic arterial pressure < 90 mmHg, or evidence of new-onset cardiac arrhythmias.

### Randomisation

All patients who passed the spontaneous breathing trial were prepared for extubation and randomly assigned in a 1:1 ratio to receive either HFNC or NIV. Randomisation was performed using a computer-generated randomisation table derived from www.randomization.com. This process was performed by the principal investigator (S.T.). The device assignments were placed in concealed envelopes, which were labelled with a sequential number. The other investigators, the patients, the patients’ relatives, the attending physicians, and the nurses were all blinded to the study assignment. The concealed envelope was opened after the patient was enrolled with a signed informed consent.

### Procedures

After extubation, the patient underwent HFNC or NIV depending on the results of randomisation. For the HFNC group, a High-flow: Optiflow® system was used. The device setting was started at an oxygen flow rate of 30 L/min at a temperature of 37 °C. The flow rate was titrated by 5 L/min every 10 min to achieve patient demand while maintaining arterial blood pH > 7.35 and PaCO_2_ < 60 mmHg. The maximum flow rate was not more than 50 L/min. The fraction of inspired oxygen (FiO_2_) was started at 40% and adjusted to maintain the patient’s oxygen saturation at 92% or more. The HFNC device was continuously applied to the patient for 24 h. If the patient remained clinically stable, the setting was reduced according to the patients' status.

For the NIV group, a Drager Carina noninvasive ventilator with a full facemask interface was used. The NIV interface size was selected to cover the patient’s mouth and nose to minimize air leakage. The NIV interface was hand held by a physician while adjusting the NIV pressure setting for 10 to 15 min. Once the patient was familiar with NIV assistance, a strapped mask was applied that was tight enough to minimize air leakage while avoiding patient discomfort. The bilevel positive airway pressure (BiPAP) mode was used beginning with an inspiratory positive pressure (P_inp_) of 8 cmH_2_O and expiratory positive pressure (P_exp_) of 5 cmH_2_O. The FiO_2_ was started at 40% and adjusted to maintain the patient’s oxygen saturation at 92% or more. The P_inp_ was titrated by 2 cmH_2_O every 10 min to achieve patient demand while maintaining arterial blood pH > 7.35 and PaCO_2_ < 60 mmHg. The maximum P_inp_ was limited to 20 cmH_2_O. The device was continuously applied to the patient for 24 h, except during airway secretion clearance. If the patient remained clinically stable for 24 h, the duration and setting of NIV use was reduced according to the patient’s status. Standard intensive care unit monitoring, including electrocardiography, oxygen saturation, and exhaled PaCO_2_, were continuously performed. Arterial blood gas analysis was performed 1 h after applying the device.

### Outcomes

The primary outcome of this study was the rate of reintubation at 72 h after planned extubation. Reintubation was performed if patients developed one of the following conditions: (i) respiratory or cardiac arrest, (ii) respiratory pauses with loss of consciousness or gasping for air, (iii) Glasglow coma score < 8, (iv) massive aspiration or persistent inability to remove respiratory secretions, (v) heart rate < 50 or > 140 beats/minute with signs of poor tissue perfusion, (vi) severe hemodynamic instability unresponsive to treatment, (vii) extubation failure unresponsive to treatment for 30 min, and (viii) reintubation because of other reasons, such as urgent surgery. The reintubation was performed by the patient’s attending physician. The definition of extubation failure included (1) respiratory rate > 35 breaths/minute, (2) oxygen saturation < 90% or PaO_2_ < 80 mmHg despite receiving FiO_2_ > 50%, (3) respiratory acidosis with pH < 7.35 or PaCO_2_ > 50 mmHg or an increase of 20% from baseline. Patients with these findings were treated with the assigned device adjusted to its upper limit of the protocol. If the extubation failure did not improve, the patient was switched to the other device as the rescue therapy. We limited rescue therapy to 30 min under close observation. Patients who continued to deteriorate after changing the device were reintubated. Patients who required reintubation or died before reintubation were also described as extubation failure. The secondary outcomes were reintubation rates at 7 days, at 28 days after extubation, rate of extubation failure at 72 h, ICU mortality rate, hospital mortality rate, and the 28-day mortality rate.

### Statistical analysis

From our experience with critically ill medical patients on NIV, we observed that 40% experienced extubation failure [28]. We hypothesized that HFNC would reduce the reintubation rate by an absolute 20% reduction. Enrolment of at least 102 participants per group would provide at least 90% power to detect a difference of 20% in the primary outcome between the two groups at a two-sided alpha error of 0.05.

Continuous variables were presented using either mean (standard deviation) or median (range) and analysed by *t* test or Wilcoxon rank-sum test, when suitable. Categorical variables were presented using frequency and percentage. Chi-squared test or Fisher’s exact test was used as appropriate. The primary and secondary outcomes were analysed by Chi-squared test and presented as relative risk with a 95% confidence interval (CI). The Kaplan–Meier curve was used to assess time from extubation to reintubation, followed by a comparison using the log-rank test at 72 h. For mortality analysis, the 28-day mortality was calculated from the date of extubation. All primary and secondary outcome analyses were based on the intention-to-treat principle and a *P* value < 0.05 was considered statistically significant. Data were analysed using SPSS Statistics version 18 (SPSS.Inc., Chicago, IL). This study was registered on the ClinicalTrials.gov database (identification number: NCT03246893).

## Results

A total of 1016 patients were admitted to the medical ICU between October 1, 2017 and October 31, 2019. Of these, 930 patients required mechanical ventilation and were assessed for eligibility. A total of 222 patients provided consent and underwent randomisation. One hundred and twelve patients were randomly assigned to the HFNC group and 110 patients were assigned to the NIV group (Fig. 1). Demographic characteristics, including age, gender, underlying conditions, and severity scores were well-matched between groups (Table 1 and Additional file 1). The main cause of intubation was shock-related respiratory failure; (64/112 [57.1%] in the HFNC group and 61/110 [55.5%] in the NIV group). Pneumonia was the leading cause of infection (140 patients; 63%) and septic shock was diagnosed in 173 patients (77.9%). The median duration of intubation was 5 days in both groups. The number of patients at increased risk for reintubation, including those with aged > 65 years, with acute physiology and chronic health evaluation (APACHE) II score at the day of extubation > 12, and mechanical ventilation > 7 days, was similar between groups (Table 1). Patients in the NIV group received the maximum P_inp_ at a median of [IQR] 12 [10–14] cmH_2_O, P_exp_ at a median of 5 [5–8] cmH_2_O and FiO_2_ was 40% [40–60], sequentially. In the HFNC group, the maximum flow rate was 40 [40–45] L/min and the FiO_2_ was 40% [40–60].

**Table 1:** Baseline characteristics of the patients

Baseline characteristic	NIV (*N* = 110)	HFNC (*N* = 112)	*P*
Age, mean (SD), year	63.0 (17.5)	62.6 (1803)	0.85
Age > 65 years, No. (%)	58 (52.7)	60 (53.6)	0.90
Male gender, No. (%)	61 (55.5)	60 (53.6)	0.78
Comorbidities, No. (%)			
Hypertension	77 (70.0)	67 (59.8)	0.15
Diabetes mellitus	53 (48.2)	43 (38.4)	0.18
Chronic kidney disease	42 (38.2)	30 (26.8)	0.10
Congestive heart failure	5 (4.5)	7 (6.3)	0.61
Chronic obstructive pulmonary disease	7 (6.4)	3 (2.7)	0.32
Body mass index, mean (SD), kg/m^2^	24.1 (4.7)	23.6 (4.7)	0.47
Body mass index ≥ 30 kg/m^2^, No. (%)	15 (13.6)	12 (10.7)	0.65
Duration of intubation, median (IQR), day	5 (3–9)	5 (3–8)	0.51
Duration of intubation > 7 days, No. (%)	43 (39.1)	37 (33)	0.42
APACHE II score^a^ at extubation, median (IQR)	15 (11–16)	15 (11–18)	0.60
SOFA score^b^ at extubation, median (IQR)	4 (2–6)	3 (2–5)	0.35
Source of infection, No. (%)			
Pneumonia	75 (68.2)	65 (58)	0.15
Intra-abdominal infection	11 (10.0)	14 (12.5)	0.56
Urinary tract infection	10 (9.1)	8 (7.1)	0.60
Soft tissue infection	4 (3.6)	8 (7.1)	0.37
Bacteraemia	8 (7.3)	15 (13.4)	0.20
Others	2 (1.8)	2 (1.8)	0.99
Causes of intubation, No. (%)			
Shock-related respiratory failure	61 (55.5)	64 (57.1)	0.80
Hypoxic respiratory failure	45 (40.9)	39 (34.8)	0.43
Hypercapnic respiratory failure	4 (3.6)	9 (8.0)	0.25
Septic shock, No. (%)	88 (80)	85 (75.9)	0.57
Weaning method, No. (%)			
Pressure support	86 (78.2)	76 (67.9)	0.11
T-piece	24 (21.8)	36 (32.1)	0.11
ABG prior to extubation, mean (SD)			
pH	7.45 (0.06)	7.45 (0.05)	0.84
PaO_2_, mmHg	138.4 (37.2)	139.5 (41.2)	0.83
PaCO_2_, mmHg	35.3 (6.6)	34.2 (6.8)	0.23
PaO_2_:FiO_2_ ratio	348.4 (88.6)	350.8 (101.3)	0.86
Fluid accumulation*, median (IQR), litre	4.6 (1.1–7.6)	5.2 (1.1–8.8)	0.39

NIV: noninvasive ventilation; HFNC: high-flow nasal oxygen cannular; SD: standard deviation; IQR: interquartile range; kg/m^2^: kilogram per square metre; APACHE: acute physiology and chronic health evaluation; SOFA: sequential organ failure assessment; g/dL: gram per decilitre; mg/dL: milligram per decilitre; cmH_2_O: centimetre of water; mL/kg: millilitre per kilogram; ABG: arterial blood gas analysis; PaO_2_: partial pressure of oxygen in arterial blood; PaCO_2_: partial pressure of carbon dioxide in arterial blood; FiO_2_: fraction of inspired oxygen; mmHg: millimetre of mercury

^a^APACHE II score, a severity-determining score, ranges from 0 to 71. The higher scores represent more severe disease

^b^SOFA score ranges from 0 to 24. The higher scores represent more organ failure

High risk factors for reintubation

^*^Fluid accumulation was calculated from overall fluid intake minus fluid output since patient’s admission until before extubation

**Fig. 2:**
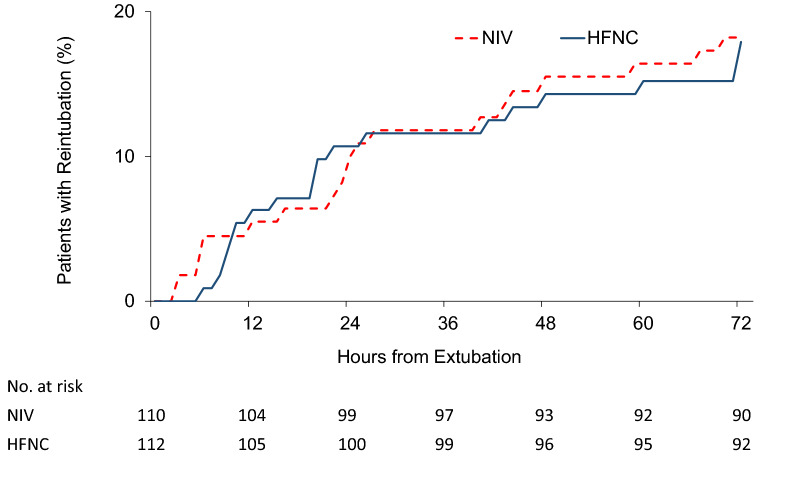
Kaplan–Meier analysis of time from extubation to reintubation. The cumulative reintubation probability was no different between HFNC group and the NIV group (log-rank *P* = 0.95). HFNC: High-flow nasal oxygen cannula; NIV: Noninvasive mechanical ventilation

The primary outcome of reintubation at 72 h occurred in 20 (17.9%) HFNC patients and 20 (18.2%) NIV patients (RR 0.99; 95% CI 0.70–1.39; *P* = 0.95). The Kaplan–Meier curve illustrated time from extubation to reintubation in Fig. 2. The rate of reintubation at 7 days (24 [21.4] vs. 30 [27.3]; RR 0.86; 95% CI 0.64–1.14; *P* = 0.31) and at 28 days (37 [33.0] vs. 40 [36.4]; RR 0.93; 95% CI 0.71–1.22; *P* = 0.6) were similar. The rate of reintubation in ICU was also not different (33 [29.5] vs. 37 [33.6]; RR 0.91; 95% CI 0.69–1.19; *P* = 0.5). Extubation failure at 72 h occurred in 27 (24.1%) HFNC patients and 34 (30.9%) NIV patients (RR 0.85; 95% CI 0.64–1.12; *P* = 0.26). The overall extubation failure in ICU was not significantly different (37 [33%] vs. 47 [42.7%]; RR 0.82; 95% CI 0.63–1.06; *P* = 0.14).

**Fig. 3:**
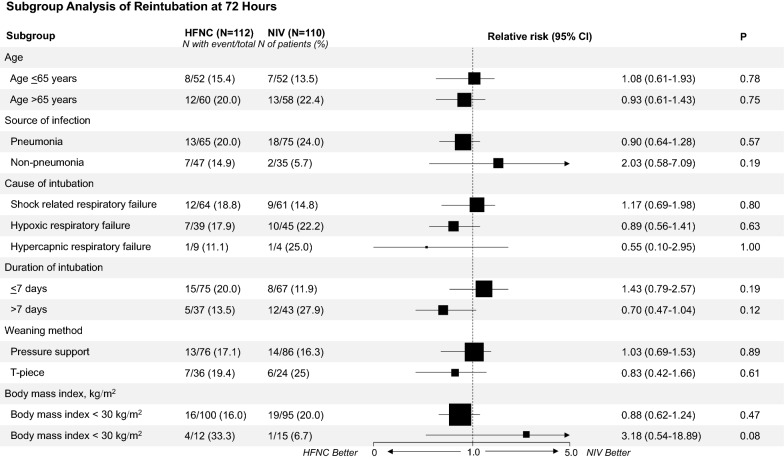
Risk of reintubation at 72 h after extubation, according to subgroup. Subgroup analysis according to six patients’ characters. The location of square represents the relative risk and the size of square reflects the relative numbers in each subgroup. The horizontal bars represent 95% confidence intervals

Among the 34 patients in the NIV group that developed extubation failure, 14 patients (12.7%) were successfully switched to HFNC without reintubation, while seven patients (6.3%) of 27 patients who developed extubation failure in the HFNC group responded to NIV. There was no difference in the interval from extubation to device change (median [IQR] 24 [20–53] h HFNC and 23 [19–41] h NIV; *P* = 0.54). ICU mortality rate was not significantly different; HFNC 6 [5.4%] vs. NIV 12 [10.9%]; (RR 0.72; 95% CI 0.51–1.03; *P* = 0.13). The 28-day mortality (8 [7.1] vs. 10 [9.1]; RR 0.88; 95% CI 0.57–1.37; *P* = 0.59) and hospital mortality (24 [21.4] vs. 26 [23.6]; RR 0.94; 95% CI 0.69–1.28; *P* = 0.69) also did not differ between groups (Table 2). Subgroup analysis for the relative risk of reintubation and extubation failure at 72 h is shown in Fig. 3 and Additional file 2: Table S2. There was no significant differences between the HFNC and NIV in the rate of reintubation and extubation failure at 72 h among patients who were weaning by pressure support or T-piece trial (see Additional file 2).

**Table 2:** Primary and secondary outcomes

Outcomes	NIV (*N* = 110)	HFNC (*N* = 112)	Relative risk (95% CI)	*P*
Primary outcomes, No. (%)				
Reintubation at 72 h	20 (18.2)	20 (17.9)	0.99 (0.70–1.39)	0.95
Secondary outcomes, No. (%)				
Reintubation at 7 days	30 (27.3)	24 (21.4)	0.86 (0.64–1.14)	0.31
Reintubation at 28 days	40 (36.4)	37 (33.0)	0.93 (0.71–1.22)	0.6
Reintubation in ICU	37 (33.6)	33 (29.5)	0.91 (0.69–1.19)	0.5
Extubation failure at 72 h	34 (30.9)	27 (24.1)	0.85 (0.64–1.12)	0.26
Extubation failure in ICU	47 (42.7)	37 (33.0)	0.82 (0.63–1.06)	0.14
Mortality				
ICU mortality	12 (10.9)	6 (5.4)	0.72 (0.51–1.03)	0.13
28-day mortality	10 (9.1)	8 (7.1)	0.88 (0.57–1.37)	0.59
In-hospital mortality	26 (23.6)	24 (21.4)	0.94 (0.69–1.28)	0.69
Length of stay, median (IQR), days				
ICU length of stay	10 (6–22)	10 (6–19)		0.68
Hospital length of stay	24 (14–44)	28 (19–48)		0.23

There was no difference in the duration of device usage (median [IQR] 24 [20–55] h HFNC and 24 [19–42] h NIV; *P* = 0.18) and duration of reintubation. The leading cause of reintubation was hypoxia (17.9% in HFNC vs. 19.1% in NIV; *P* = 0.81). Other causes for reintubation included hemodynamic instability, cardiac arrest, the need for urgent surgery, and the inability to clear secretions (Table 3). The median [IQR] ICU length of stay was similar between groups (10 [6–19] days in the HFNC group vs. 10 [6–22] days in the NIV group, *P* = 0.68). The duration of hospitalisation was not different either (28 [19–48] days in the HFNC group vs. 24 [14–44] days in the NIV group; *P* = 0.23).

**Table 3:** Duration of device usage and causes of reintubation

Variables	NIV (*N* = 110)	HFNC (*N* = 112)	Relative risk (95% CI)	*P*
Duration of device usage, median (IQR), h	24 (19–42)	24 (20–55)		0.18
Interval from extubation to reintubation, median (IQR), h	67 (23–139)	54 (19–282)		0.90
Device protocol compliance rate	105 (95.5)	107 (95.5)	0.99 (0.49–2.01)	0.98
Causes of reintubation, No. (%)				
Hypoxia	21 (19.1)	20 (17.9)	0.96 (0.69–1.34)	0.81
Hemodynamic instability	6 (5.5)	4 (3.6)	0.82 (0.48–1.38)	0.50
Cardiac arrest	4 (3.6)	4 (3.6)	0.99 (0.49–2.01)	0.98
Urgent surgery	2 (1.8)	1 (0.9)	0.74 (0.33–1.67)	0.55
Inability to clear secretions	1 (0.9)	4 (3.6)	2.51 (0.43–14.57)	0.18
Altered mental status	1 (0.9)	4 (3.6)	2.51 (0.43–14.57)	0.18
Others	2 (1.8)	1 (0.9)	0.65 (0.38–1.17)	0.50

Calculated using patients who required reintubation (37 in the NIV group and 33 in the HFNC group)

*Calculated using patients who required a device change (20 in the NIV group and nine in the HFNC group)

## Discussion

In this RCT of patients who recovered from sepsis/septic shock, we found no statistically significant differences in the rates of reintubation or extubation failure between the HFNC and NIV treatment groups. Twenty-eight day and in-hospital mortality rates in the two groups were not significantly different.

Extubation failure increases the risk of mortality and prolongs ICU stays in high risk patients [3–7]. In our sepsis patients, we found that both HFNC and NIV worked equally well. We observed that reintubation rates in both groups were similar, but the proportion of patients with extubation failure was not significantly higher with NIV than with HFNC. This finding is consistent with a study by Hernandez et al. in high-risk medical and surgical patients, where reintubation rates at 72 h were 19.1% NIV and 22.8% HFNC [15], while in ours, 18.2% and 17.9%. However, in the Hernandez study participants were a mix of surgical and medical patients with 10% having chronic obstructive pulmonary disease (COPD). All patients in our study had medical sepsis, mainly from pneumonia, with a very low proportion of COPD patients. While NIV has been proven to be beneficial in COPD or cardiogenic pulmonary oedema, the low proportion of patients with these two comorbidities may have reduced the benefit of NIV measured in our study population.

A multicentre study in France compared HFNC with NIV vs. HFNC alone in high-risk patients. This alternative mode of treatment resulted in better outcomes and a lower reintubation rate at day 7 (11.8% in HFNC and NIV vs. 18.2% in HFNC alone) [29]. However, nearly, half of the patients in that study had chronic heart disease and more than one-fifth had COPD. This suggests that HFNC and NIV are effective in preventing reintubation, but their effects depend on the patients’ condition. Nevertheless, currently there is no single protocol that applies to all patients, and alternative techniques or switching therapy may be beneficial.

Different weaning methods may be associated with different extubation outcomes. In our study, the proportion of patients who passed spontaneous breathing trial by pressure support was not significantly higher in the NIV group than in the HFNC group (78.2% vs. 67.9%; *P* = 0.11), which raises concern for unequal severity in the different treatment groups. However, data from a randomized trial showed that the reintubation rate among patients who passed spontaneous breathing trial by pressure support was not different from those who passed spontaneous breathing trial using the T-piece method [5]. Furthermore, our subgroup analysis of weaning methods indicated that there was no significant difference between groups in the rate of reintubation and extubation failure at 72 h (Additional file 2: Table S2). ICU mortality was not significantly different in the HFNC group (5.4%) vs. the NIV group (10.9%) (*P* = 0.13). Post-ICU discharge, the hospital mortality increased from 5.4 to 21.4% in the HFNC group, and from 10.9 to 23.6% in the NIV group. This additional mortality could have multiple causes. From our previously study, 28.6% of septic shock patients who had shock reversal died later in the hospital. Multi-organ failure and hospital-acquired pneumonia were the leading causes of death, followed by other infection and noninfectious complication [30]. Applying HFNC or NIV immediately after extubation could reduce reintubation and may prevent hospital-acquired pneumonia. This may result in an improved overall septic shock treatment outcome.

Our study had limitations. First, the primary outcome rate of NIV in our study was 18.2%, which was lower than the expected rate of 40%. The primary outcome rate of HFNC was 17.9%, which was closer to the expected rate of 20%. The lower rates of the primary outcome in the control group caused this study to be underpowered to detect the differences between these two devices. However, the minimal differences in outcomes are unlikely to change with a larger sample size. Second, because of the nature of the devices, the clinicians could not be blinded and this may have introduced some bias. However, we tried to minimise this bias by proposing criteria for extubation failure and reintubation, which all of the attending physicians followed. The patients were reintubated when they met these criteria, regardless of the devices. Third, this was a single-centre study that only enrolled patients with medical sepsis/septic shock. As such, our results may not be generalizable to other patient populations. A multicentre study enrolling a larger population is required to more precisely determine the benefits of HFNC or NIV after extubation of sepsis/septic shock patients.

## Conclusions

In conclusion, HFNC applied immediately post-extubation did not confer statistically significant protection over NIV to prevent reintubation and extubation failure in patients who had recovered from sepsis/septic shock.

## Supplementary Information

**Additional file 1: Table S1.** Physiologic parameters of the patients.

**Additional file 2: Table S2.** Subgroup analysis of the patients’ baseline characters.

## Data Availability

Researchers may contact the corresponding author at surat.ton@mahidol.ac.th for data sharing requests, after approval of a planned analysis protocol. The anonymized participant data will be made available within 3 months after the publication of the article. The study protocol and statistical analysis plan are available as an appendix.

## Abbreviations

HFNC: High-flow-oxygen via nasal cannula
NIV: Non-invasive mechanical ventilation
ICU: Intensive care unit
RCT: Randomised controlled trial
PaO_2_: FiO_2_ ratio, partial pressure of oxygen in arterial blood to fraction of inspired oxygen
PEEP: Positive end expiratory pressure
PaCO_2_: Partial pressure of carbon dioxide
P_inp_: Inspiratory positive pressure
