# Supplementary material for: High-flow nasal oxygen cannula vs. noninvasive mechanical ventilation to prevent reintubation in sepsis: a randomized controlled trial
Source: Ann Intensive Care. 2021 Sep 14;11:135. doi: 10.1186/s13613-021-00922-5 (PMC8439370; doi:10.1186/s13613-021-00922-5)
Supplement: Supplementary file 1 — Additional file 1: Table S1. Physiologic parameters of the patients. [file 13613_2021_922_MOESM1_ESM.docx]

| **Table S1: Physiologic parameters of the Patients** | | | |
| --- | --- | --- | --- |
| **Baseline characteristic** | **NIV**  **(N = 110)** | **HFNC**  **(N = 112)** | **P** |
| APACHE II score^a^, median (IQR) |  |  |  |
| At Admission | 26 (22-32) | 25 (21-31) | 0.85 |
| At Extubation | 15 (11-16) | 15 (11-18) | 0.50 |
| SOFA score^b^, median (IQR) |  |  |  |
| At Admission | 9 (8-12) | 10 (7-12) | 0.95 |
| At Extubation | 4 (2-6) | 3 (2-5) | 0.16 |
| Vital signs prior to extubation, mean (SD) |  |  |  |
| Heart rate, per min | 90 (18) | 93 (15) | 0.11 |
| Respiratory rate, per min | 20 (4) | 21 (4) | 0.41 |
| Mean arterial blood pressure, mmHg | 96 (15) | 94 (18) | 0.52 |
| Temperature, ^O^c | 36.8 (0.6) | 36.8 (0.6) | 0.93 |
| ABG prior to extubation, mean (SD) |  |  |  |
| pH | 7·45 (0·06) | 7·45 (0·05) | 0.84 |
| PaO_2_, mmHg | 138·4 (37·2) | 139·5 (41·2) | 0.83 |
| Oxygen saturation, % | 99.1 (1.4) | 98.8 (1.7) | 0.52 |
| PaCO_2_, mmHg | 35·3 (6·6) | 34·2 (6·8) | 0.23 |
| PaO_2_:FiO_2_ ratio | 348·4 (88·6) | 350·8 (101·3) | 0.86 |
| Vital signs post extubation at 1 hour, mean (SD) |  |  |  |
| Heart rate, per min | 93 (18) | 95 (16) | 0.47 |
| Respiratory rate, per min | 21 (5) | 21 (5) | 0.70 |
| Mean arterial blood pressure, mmHg | 97 (16) | 97 (17) | 0.99 |
| Temperature, ^O^c | 36.8 (0.6) | 37.0 (0.7) | 0.16 |
| ABG post extubation at 1 hour, mean (SD) |  |  |  |
| pH | 7.43 (0.07) | 7.45 (0.06) | 0.06 |
| PaO_2_, mmHg | 132.9 (39.5) | 126.1(39.7) | 0.26 |
| Oxygen saturation, % | 99.1 (1.4) | 98.8 (1.8) | 0.19 |
| PaCO_2_, mmHg | 36.6 (8.5) | 36.2 (9.3) | 0.82 |
| PaO_2_:FiO_2_ ratio | 338.7 (97.1) | 321.8 (101.1) | 0.27 |

**Abbreviations:** NIV, noninvasive ventilation: HFNC, high-flow nasal oxygen cannular: SD, standard deviation: IQR, interquartile range: cmH_2_O, centimetre of water: mL/kg, millilitre per kilogram: ABG, arterial blood gas analysis: PaO_2_, partial pressure of oxygen in arterial blood: PaCO_2_, partial pressure of carbondioxide in arterial blood: FiO_2_, fraction of inspired oxygen: mmHg, millimetre of mercury

^a^APACHE II score, a severity-determining score, ranges from 0 to 71. The higher scores represent more severe disease

^b^SOFA score ranges from 0 to 24. The higher scores represent more organ failure
