# Supplementary material for: High-flow nasal oxygen cannula vs. noninvasive mechanical ventilation to prevent reintubation in sepsis: a randomized controlled trial
Source: Ann Intensive Care. 2021 Sep 14;11:135. doi: 10.1186/s13613-021-00922-5 (PMC8439370; doi:10.1186/s13613-021-00922-5)
Supplement: Supplementary file 2 — Additional file 2: Table S2. Subgroup analysis of the patients’ baseline characters. [file 13613_2021_922_MOESM2_ESM.docx]

| **Table S2: Subgroup analysis of the patients’ baseline characters** | | | | | | | | |
| --- | --- | --- | --- | --- | --- | --- | --- | --- |
| Subgroup n/total (%) | Extubation failure at 72 h | | | | Reintubation at 72 h | | | |
|  | NIV (N=110) | HFNC (N=112) | Relative risk (95%CI) | P | NIV (N=110) | HFNC (N=112) | Relative risk (95% CI) | P |
| Age |  |  |  |  |  |  |  |  |
| Age <65 years | 14/52 (26.9) | 11/52 (21.2) | 0.86 (0.57-1.30) | 0.49 | 7/52 (13.5) | 8/52 (15.4) | 1.08 (0.61-1.93) | 0.78 |
| Age >65 years | 20/58 (34.5) | 16/60 (26.7) | 0.83 (0.57-1.21) | 0.36 | 13/58 (22.4) | 12/60 (20.0) | 0.93 (0.61-1.43) | 0.75 |
| Source of infection |  |  |  |  |  |  |  |  |
| Pneumonia | 29/75 (38.7) | 17/65 (26.2) | 0.78 (0.57-1.05) | 0.12 | 18/75 (24.0) | 13/65 (20.0) | 0.90 (0.64-1.28) | 0.57 |
| Non-pneumonia | 5/35 (14.3) | 10/47 (21.3) | 1.34 (0.63-2.88) | 0.42 | 2/35 (5.7) | 7/47 (14.9) | 2.03 (0.58-7.09) | 0.19 |
| Cause of intubation |  |  |  |  |  |  |  |  |
| Shock related respiratory failure | 15/61 (24.6) | 17/64 (26.6) | 1.06 (0.69-1.61) | 0.80 | 9/61 (14.8) | 12/64 (18.8) | 1.17 (0.69-1.98) | 0.80 |
| Hypoxic respiratory failure | 17/45 (37.8) | 9/39 (23.1) | 0.74 (0.50-1.09) | 0.15 | 10/45 (22.2) | 7/39 (17.9) | 0.89 (0.56-1.41) | 0.63 |
| Hypercapnic respiratory failure | 2/4 (50.0) | 1/9 (11.1) | 0.30 (0.07-1.31) | 0.20 | 1/4 (25.0) | 1/9 (11.1) | 0.55 (0.10-2.95) | 1.00 |
| Duration of intubation |  |  |  |  |  |  |  |  |
| <7 days | 17/67 (25.4) | 20/75 (26.7) | 1.04 (0.69-1.55) | 0.86 | 8/67 (11.9) | 15/75 (20.0) | 1.43 (0.79-2.57) | 0.19 |
| >7 days | 17/43 (39.5) | 7/37 (18.9) | 0.66 (0.45-0.96) | 0.05 | 12/43 (27.9) | 5/37 (13.5) | 0.70 (0.47-1.04) | 0.12 |
| Weaning method |  |  |  |  |  |  |  |  |
| Pressure support | 27/86 (31.4) | 18/76 (23.7) | 0.84 (0.62-1.13) | 0.27 | 14/86 (16.3) | 13/76 (17.1) | 1.03 (0.69-1.53) | 0.89 |
| T-piece | 7/24 (29.2) | 9/36 (25.0) | 0.88 (0.45-1.72) | 0.72 | 6/24 (25) | 7/36 (19.4) | 0.83 (0.42-1.66) | 0.61 |
| Body mass index, kg/m^2^ |  |  |  |  |  |  |  |  |
| Body mass index < 30 kg/m^2^ | 30/95 (31.6) | 22/100 (22.0) | 0.79 (0.59-1.06) | 0.13 | 19/95 (20.0) | 16/100 (16.0) | 0.88 (0.62-1.24) | 0.47 |
| Body mass index > 30 kg/m^2^ | 4/15 (26.7) | 5/12 (41.7) | 1.38 (0.61-3.12) | 0.41 | 1/15 (6.7) | 4/12 (33.3) | 3.18 (0.54-18.89) | 0.08 |
